# A Systematic Review of the Clinimetric Properties of Habitual Physical Activity Measures in Young Children with a Motor Disability

**DOI:** 10.1155/2012/976425

**Published:** 2012-08-09

**Authors:** Stina Oftedal, Kristie L. Bell, Louise E. Mitchell, Peter S. W. Davies, Robert S. Ware, Roslyn N. Boyd

**Affiliations:** ^1^Queensland Cerebral Palsy & Rehabilitation Research Centre, School of Medicine, The University of Queensland, Brisbane, QLD 4029, Australia; ^2^Children's Nutrition Research Centre, School of Medicine, The University of Queensland, Brisbane, QLD 4029, Australia; ^3^Queensland Children's Medical Research Institute, The Royal Children's Hospital, Brisbane, QLD 4029, Australia; ^4^The Royal Children's Hospital, Herston, Brisbane, QLD 4029, Australia; ^5^School of Population Health, The University of Queensland, Brisbane, QLD 4006, Australia

## Abstract

*Aim*. To identify and systematically review the clinimetric properties of habitual physical activity (HPA) measures in young children with a motor disability. *Method*. Five databases were searched for measures of HPA including: children aged <6.0 years with a neuromuscular disorder, physical activity defined as “bodily movement produced by skeletal muscles causing caloric expenditure”, reported HPA as duration, frequency, intensity, mode or energy expenditure, and evaluated clinimetric properties. The quality of papers was assessed using the COSMIN-checklist. A targeted search of identified measures found additional studies of typically developing young children (TDC). *Results*. Seven papers assessing four activity monitors met inclusion criteria. Four studies were of good methodological quality. The Minimod had good ability to measure continuous walking but the demonstrated poor ability to measure steps during free-living activities. The Intelligent Device for Energy Expenditure and Activity and Ambulatory Monitoring Pod showed poor ability to measure activity during both continuous walking and free-living activities. The StepWatch showed good ability to measure steps during continuous walking in TDC. *Interpretation*. Studies assessing the clinimetric properties of measures of HPA in this population are urgently needed to allow assessment of the relationship between HPA and health outcomes in this group.

## 1. Introduction

Habitual physical activity (HPA) is an established determinant of health in children and is required for healthy development, including the growth of bone and muscle mass, improved balance and motor skills, maintaining a healthy weight and improved psychological wellbeing [1, 2]. Limited evidence suggests young children with motor disorders are less physically active than their typically developing peers [3]. Consequently they may be at risk of suboptimal growth and development in addition to the development of secondary conditions such as chronic pain, fatigue, and low bone density which can lead to diminished bone health [4, 5]. Australian physical activity guidelines state children aged from one to five years should be physically active for three hours throughout the day and should not be sedentary, restrained, or kept inactive, for more than one hour at a time, with the exception of sleeping [2]. Studies investigating the link between HPA and health outcomes for children with motor impairments have not been conducted. These studies are urgently needed to (i) determine the HPA patterns and intensities of children with motor disabilities, (ii) support the importance of physical activity promotion and inactivity prevention, (iii) determine the dose-response relationship between physical activity and health outcomes, and (iv) allow assessment of the effectiveness of interventions aimed at increasing HPA.

Physical activity is defined as “any bodily movements using skeletal muscles that results in energy expenditure” [6]. The International Classification of Functioning, Disability and Health (ICF) further divides physical activity into the concepts of “performance” and “capacity” [7]. Performance is defined as the execution of an activity in the natural environment. This is distinct from capacity which refers to a child's maximal ability to perform an activity in an ideal or standardised environment. Performance and capacity to complete a task (e.g., jumping) can be determined by observing the child undertake that specific task within a limited time frame in their natural and ideal environment, respectively. HPA, on the other hand, describes a child's typical daily activity pattern and hence includes their performance of a multitude of activities which necessitates measurement across multiple days [8]. The variables of frequency (how often a child does an activity), intensity (how hard a child works to do the activity), duration (how long a child does an activity), and mode (what the child is doing) are used to characterise HPA patterns, while activity-related energy expenditure (AEE) is used as a summary variable of all the other indicators [9, 10]. 

Direct observation is considered the gold standard for physical activity classification, while doubly labelled water in combination with resting energy expenditure is considered the gold standards for calculating AEE [10]. Measures of HPA include both objective and subjective methods [11]. Objective measures include heart rate monitors, accelerometers, and pedometers. Subjective methods include self- or proxy reports, interviews, and diaries. The measurement of HPA in typically developing children (TDC) has received a great deal of attention in the last decade, primarily due to a concern over the increasing rate of overweight and obesity in childhood [12]. A systematic review of pedometers and accelerometers has identified moderate-to-good evidence of validity and strong evidence of reliability for the ActiGraph accelerometer in preschool-aged TDC [13]. Children with motor impairments may not be walking but instead cruising, crawling, bottom shuffling, rolling, use walking aids or a wheelchair as means of locomotion, and those who are walking may have different gait patterns and speed of movement than TDC [14]. This variation in movement patterns necessitates validation of motion sensors specifically for this population. 

Systematic reviews of measures of physical activity in children and adolescents with cerebral palsy (CP) have been conducted [15, 16]. Capio et al. [15] included children with CP from birth to 18 years and concluded that the subjective measures of the Activities Scale for Kids-Performance Version (ASKp) and the Children's Assessment of Participation and Enjoyment (CAPE) are the only measures with established validity and reliability in children with physical disabilities from the age of six years. The review by Clanchy et al. [16] included children with CP aged 10–18 years and identified the CAPE as the measure with the strongest evidence of reliability and validity. It was also noted that no measures have been assessed in children with more severe disability who are wheelchair dependent. This paper did not include the ASKp as it did not meet inclusion criteria of 60% of items relating to a domain of physical activity performance. This was done to ensure the included measures assess physical activity as traditionally defined by Caspersen et al. [6] and not “participation” which does not take into consideration the sedentary or active nature of the activity. Both reviews note the lack of HPA measures which can assess activity intensity, which limits their ability to provide meaningful comparison with physical activity guidelines, as children from the age of six years are advised to accumulate 60 minutes of moderate-to-vigorous physical activity every day [17]. This current paper aims to systematically review the clinimetric properties (validity, reproducibility, and clinical utility) of measures of HPA in children less than 6 years of age with a motor disability [6].

## 2. Method

### 2.1. Search Strategy

A systematic literature search was performed by one reviewer (SO) of the electronic bibliographic databases PubMed, CINAHL, EMBASE, SPORTDiscus, and Web of Science from their inception to September, 2011. Databases were searched using medical subject headings (MeSH) terms and text words for physical activity and disability, limiting the search to the age group <6.0 years. 

### 2.2. Inclusion and Exclusion Criteria

Measures of HPA were included which met the following criteria: (i) included children less than six years of age with a motor disability caused by neuromuscular disorders; (ii) defined physical activity as any bodily movement produced by skeletal muscles that results in caloric expenditure [6]; (iii) reported physical activity in terms of duration, frequency, intensity, or energy expenditure; (iv) questionnaires where at least 60% of items related to a domain of HPA; (v) evaluated clinimetric properties for the measurement of HPA. Studies were excluded if they were (i) not published in English, due to lack of translation services; (ii) primarily measured capacity or participation.

The title and abstract of all retrieved references were screened by the first author to exclude any papers which did not include children in the target age group or were not on the topic of physical activity measures. A second screening of abstracts was performed by two authors (SO, LM) independently to exclude those that did not include habitual physical activity measures but rather assessed capacity or participation measures. For the remaining abstracts, full papers were retrieved and screened by two authors (SO, LM). Publications which did not assess the clinimetric properties of measures of HPA in children with motor disabilities were excluded. Upon disagreement between the two reviewers, a third reviewer (KB) reviewed the publication in question. The reference lists of included papers and relevant reviews were screened, and electronic author and citation tracking were performed when available, to identify relevant publications not identified by the initial search strategy. For measures where clinimetric properties were found in children less than six years of age with a motor disability, a further search identified any evidence in typically developing children in the same age group. 

### 2.3. Data Extraction and Quality Review

The COSMIN (COnsensus-based Standards for the selection of health Measurement INstruments) checklist [18] was used to rate the methodological quality of the study designs used to evaluate the clinimetric properties validity and reproducibility for measures of HPA. The COSMIN was developed through an international Delphi study to assess the methodological quality of studies on measurement properties of health-related patient-reported outcomes (HR-PROs) [18]; however the measurement properties are also relevant to other health-related measurement instruments. The scoring system was developed based on expert discussion and testing on 46 articles identified by a systematic review and uses a “worst score counts” algorithm [19]. Each item within a specific measurement property is rated individually as “excellent” (+++), “good” (++), “fair” (+), or “poor” (−) and an overall score is given by taking the lowest score of any of the items within the assessment of the measurement property. Measurement properties included in this paper were measurement error (COSMIN box C), hypothesis testing (COSMIN box F), and criterion validity (COSMIN box H). The methodological quality rating of the papers was separated into study design and statistical analysis.

### 2.4. Rating of Study Design

The study design for the assessment of measurement properties was rated as having “excellent” quality if all relevant items within a given checklist scored “excellent.” Study design was rated as “good” if information for some items was not complete and therefore could not be scored “excellent,” though it could be assumed these were of “good” quality. A “fair” quality rating was given if there were minor flaws in the design. If the results were not to be trusted because of major flaws in the design, a study is rated as “poor” [19]. To assess if there were any important flaws in the design or methods of the study (COSMIN Box H, item five), items from the systematic review of activity monitors in TDC were used [13] as a guide in addition to whether or not the testing protocol included free-living activities.

One modification was made to the original COSMIN checklist. Proposed sample size requirements [20] were applied in the clinimetric properties section instead of the study design section. The decision to separate study design and sample size requirements was made as sample size does not determine study design quality as such but does affect the statistical power available to detect a significant result.

### 2.5. Statistical Methods and Clinimetric Properties of HPA Measures

For the assessment of criterion validity, the COSMIN checklist accepts correlations or the area under the receiver operator characteristic (AUC-ROC) curve as “excellent” statistical methods for continuous measures [19]. For dichotomous scales sensitivity and specificity calculations are considered “excellent” measures [19]. For the assessment of concurrent validity (hypothesis testing) it is up to the reviewer to assess if the method is “appropriate” and therefore scores “excellent.” For the assessment of measurement error (agreement), Standard Error of Measurement (SEM), Smallest Detectable Change (SDC), and Limits of Agreement (LoA) calculated using the Bland-Altman method are accepted as “excellent.” According to the COSMIN a sample size of less than 30 participants is considered “poor,” 30–49 participants is considered “fair,” 50–99 participants is considered “good,” and 100 or more participants is considered “excellent” [19]. Authors were contacted to see if they had used any power calculations when determining their sample size.

Quality criteria for the clinimetric properties of measures were proposed to give a framework to help distinguish between the quality of the studies and the performance of the measurement tools [20]. Agreement, criterion validity, and construct validity were therefore scored as good (+), indeterminate (unable to assess: u/a), or poor (−) [20]. Criterion validity was considered “good” if correlation with gold standard was ≥0.70, sensitivity and specificity ≥0.70, and area under receiver operating characteristic curve ≥0.70 [20]. Construct validity was considered “good” if 75% of the results were in accordance with the hypothesis [20]. Measurement error is considered “good” if the SDC or the LoA are smaller than the minimal important change or if “authors provide convincing arguments that the agreement is acceptable” in a sample size of at least 50 participants [20]. Studies that used a “poor” statistical method scored “indeterminate” for performance of clinimetric properties. 

The assessment of clinimetric properties of HR-PRO questionnaires might be of a different nature to those assessing activity measures due to the availability of gold standards, something which is not readily available for HR-PROs as these are usually questionnaires regarding patient outcomes such as pain and quality of life [21]. The statistical methods and performance of clinimetric properties were therefore scored according to the COSMIN but were also discussed further, and a different score was given if authors presented a convincing argument that the measure was acceptable.

### 2.6. Clinical Utility

The clinical utility of measures of HPA was assessed in terms of the need for individual calibration of the equipment, the interpretability of the data output, and the need for software analysis, cost of equipment, and any required software. The feasibility of the equipment for using children aged less than six years with a motor disability in free living situations was also considered (size, weight, number of sensors, place worn, ease of correct placement, and battery life). The richness of data was also assessed in terms of what aspects of HPA (frequency, duration, intensity, mode, and energy expenditure) it was able to assess in children with a motor disability. 

### 2.7. Overall Level of Evidence

The overall level of evidence for the clinimetric properties of measures of HPA in young children with motor disabilities was determined as a whole by assessing the methodological quality of study design and statistical methods used in the assessment of HPA measures using the “worst score counts” algorithm, agreement between studies on the quality of clinimetric properties of these HPA measures, and the number of studies available for each HPA measure. 

## 3. Results

**Figure 1:**
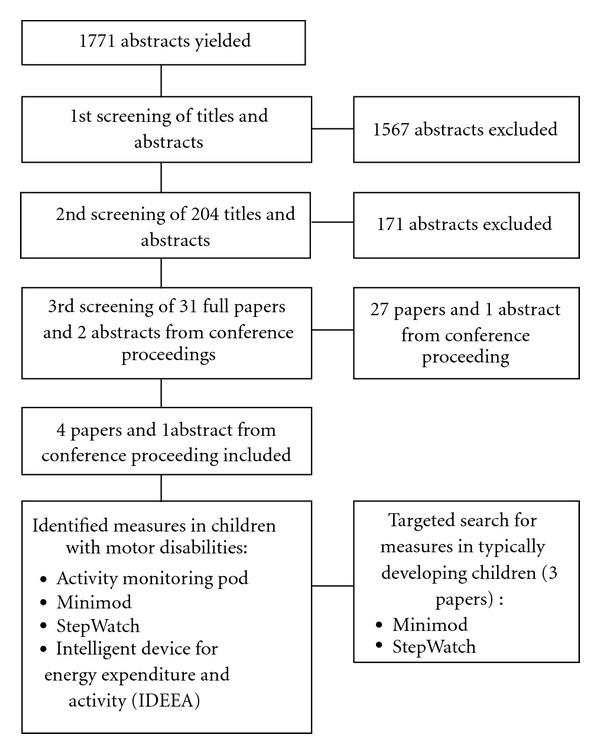
Flow diagram for search strategy.

Description of the results of the search strategy can be seen in Figure 1. The initial search yielded 1771 titles, and abstracts after duplicates were deleted. The initial screening by titles and abstracts excluded 1567 titles, and the second screening excluded a further 171 papers. Thirty-one full papers and two abstracts from conference proceedings were further reviewed. Four papers and one abstract from conference proceedings on the clinimetric properties of four measures of HPA in children with motor disabilities were identified which included children aged less than six years in their sample. None of the studies focused exclusively on children aged less than six years, which led to the inclusion of studies with a wide age range (4–18 years).

The characteristics of the studies are detailed in Table 1. Evidence of clinimetric properties was found for three accelerometer-based activity monitors [22–26] and one pedometer using inertial sensors [23]. A paper by Kuo et al. [23] and an abstract from conference proceedings by Brandes et al. [22] were identified for the Minimod pedometer (DynaPort McRoberts, Hague, Netherlands). The Activity Monitoring Pod-331 pedometer which uses inertial sensors (AMP; Dynastream Innovations, Alberta, Canada) was also assessed in the paper by Kuo et al. [23]. Papers by Stevens et al. [24] and McDonald et al. [25] were identified for the StepWatch pedometer (Orthocare Innovations, WA, USA). A paper by Aviram et al. [26] assessed the Intelligent Device for Energy Expenditure and Activity (IDEEA; Minisun, CA, USA). Only McDonald et al. [25] assessed children with Duchenne muscular dystrophy (DMD); others assessed children with CP who were able to walk with or without aids (Gross Motor Function Classification System levels I–III) [22–24, 26]. None of the study samples included children who did not walk as their main means of locomotion.

All of the studies of children with motor disabilities included TDC as a reference group [22–26]. Sample sizes ranged from 17 to 27 children with motor disabilities and 7–27 in the TDC reference group. All authors were contacted to clarify the proportion of children in their study who were under the age of six. The study by Brandes et al. [22] on the use of the Minimod included one child (5%) with CP aged five years old and four children (20%) in the TDC sample aged three to five years. The study by Aviram et al. [26] included nine children (43%) aged four to five years with three children in each GMFCS category (I–III). The authors did not supply the number of TDC aged five years, but the youngest of the seven children in the TDC group was 5 years and 8 months old [26]. In the study by Kuo et al. [23] it was estimated that a maximum of two children in the CP (11%) and TDC (10%) group were less than six years old, although authors were not able to readily access information to confirm this. It is not known how many children were under the age of six in the study by Stevens et al. [24] and McDonald et al. [25]. The targeted search for studies reporting on the clinimetric properties of the identified measures in young TDC identified a further three studies assessing the Minimod [27] and the StepWatch [28, 29]. The sample size in the three studies ranged from 20 to 162 children, and ages ranged from 2 to 16 years. The StepWatch study by Bjornson et al. [28] reported results for two-to-three-year olds (*n* = 60) and four-to-five-year olds (*n* = 62) TDC separately. The StepWatch study by Song et al. [29] used two age bands (5–7 years and 9–11 years) with ten children in each group, but the number of five year old children in the youngest group was not specified by the authors upon request. In the paper by Brandes et al. [27], authors report on the same sample of TDC (*n* = 20, four children ≤6.0 years) as in the previously mentioned abstract from conference proceedings. The full paper and abstract from conference proceedings will be rated as one paper.

### 3.1. Rating of Study Designs

Description of the study design of included papers is outlined in Table 1. None of the authors using manual step count as a criterion method reported on the accuracy of the manual step count (intra- or interrater agreement). Although it can be assumed that it is a gold standard, no evidence has been provided, and therefore the maximum attainable score for all studies according to the COSMIN checklist was “good” [19]. The study by Brandes et al. [22, 27] assessed the criterion validity of the Minimod in children with CP and TDC compared to manually counted steps and meters walked was rated “good” for study design. Authors report complete information in regarding test protocol (monitor settings, placement, data output, average steps needed for length walked, and range of steps across group) but only tests of continuous walking that may not adequately reflect free-living walking activity. The study by Kuo et al. [23] on the criterion validity of the Minimod and AMP compared to manually counted steps and measured meters walked was rated “good” on this construct. Authors report complete information on test protocol and use of a variety of structured activities: continuous walking; activity lap walking which includes walking, stopping, completing a simple task, and walking again; stair ascent and descent. 

The studies by Stevens et al. in children with CP [24], McDonald et al. in children with DMD [30], and Song et al. in TDC [29] all assessed the criterion validity of the StepWatch against manual step count and scored “poor” for study design due to providing no information on parts of their study protocol (number of steps, range of steps needed, and length walked). This does not necessarily mean the design was genuinely poor, but the lack of reporting does not enable assessment on applicability in measuring habitual walking activity and has implications for the interpretation of any reported statistics. The study by Bjornson et al. [28] assessed the StepWatch for criterion validity in TDC against manual step counts and was rated “good” for study design as they report a complete test protocol, though only assessed performance during continuous walking. 

The study by Aviram et al. [26] assessed the criterion validity of energy expenditure (EE) measured by the IDEEA compared to the gold standard of indirect calorimetry using the Cosmed (K4b2, Rome, Italy) which measured oxygen consumption (VO_2_) in five-second epochs. Authors reported complete study protocol information and assessed a range of everyday activities in addition to comfortable and fast treadmill walking and stair climbing. The study scored “good” for study design due to not providing any evidence of the Cosmed being a gold standard measurement although it can be assumed adequate [19]. Authors discuss possible limitations of the use of the Cosmed, which include a poor fitting mask, which may lead to inaccurate measurement. This study also assessed the test-retest reliability of the IDEEA and received an “excellent” rating for study design in the assessment of this construct.

The StepWatch study in children with DMD [25] used a heart rate monitor to assess its concurrent validity over four days of wear. This study was rated “fair” for study design in the assessment of this construct as an *a priori* hypothesis of the relationship between the StepWatch and heart rate was not stated, but it was possible to deduce what was expected [19]. 

### 3.2. Rating of Statistical Methods and Clinimetric Performance of Measures

The clinimetric properties measured for all the studies reviewed are outlined in Table 2. All studies apart from the study by Bjornson et al. [28] had a sample size of less than 30 children which constitutes a “poor” score on the COSMIN checklist, while the sample size of 60–62 children in each age group found in the Bjornson et al. study [28] receives “good” rating on this item. Three of the four authors who replied had not used a power calculation to determine sample size [22, 23, 26, 27], and one author stated in their reply that the recruitment of 60 children in each group (30 boys and 30 girls) was chosen as it was expected this would “increase the likelihood of approximating a normal distribution” [28]. It is not known if Stevens et al. [24] and McDonald et al. [25] used any power calculations to determine their sample size. As the COSMIN works on a “worst score counts” algorithm, the highest score possible for all but one study is therefore “poor,” but as several studies report significant results, further discussion about the clinimetric performance of measures is warranted. 

Percent agreement was used to assess the criterion validity of the Minimod in children with CP and TDC by Brandes et al. [22, 27], and the use of this method leads to a “poor” rating for statistical methods according to the COSMIN as it comes under “any other statistical method” [19]. Percent agreement is a relative measure and therefore depends on reporting of the absolute values for meaningful interpretation. Authors present rich information such as the average steps needed to walk the 20-meter track (CP children) and 160-meter track (TDC) and range of steps taken [22, 27]. It is therefore possible to see that the measurement error is small. Children with CP (*n* = 19) walked an average of 79.8 steps (min: 57; max: 126), and on average the Minimod over- or underestimated by one step (agreement = 98.7%; range: 94.1 to 101.8%) [22, 27]. TDC (*n* = 20) walked an average of 273.7 steps (min: 207; max: 377), and on average the Minimod over or underestimated by one step (agreement = 99.6%; range: 98.5 to 101.5%) [22, 27]. Children with CP walked only one-third of the steps TDC walked, and a higher likelihood that agreement occurred by chance exists. Due to good reporting of study protocol and results, percent agreement was considered an “excellent” statistical method. The Minimod showed “good” accuracy and precision for the measurement of continuous walking. A limitation of this study was the assessment of continuous walking only, and therefore the criterion validity for measuring HPA is “indeterminate.” 

The issue of use of percent agreement arises in two other studies assessing the StepWatch in TDC. Percent agreement is used by Song et al. [29] who compare steps measured and manual step count. They found a measurement error of 3 ± 1% [29] but did not report how long children walked or how many steps they took which does not allow the assessment of absolute measurement error or the likelihood that the error was low by chance. For this same study, a Pearson correlation coefficient has been reported in a separate paper [31] which by COSMIN standards was an “excellent” statistical method, but the lack of information about the study protocol still applies as correlation could have been estimated based on a small number of steps. The use of percent agreement and Pearson correlation coefficient in this study receives a “poor” rating, and evidence of criterion validity based on this study was “indeterminate.” Percent agreement was also used in the study by Bjornson et al. [28] but total steps taken are reported as ≥100 steps, and therefore it is possible to assess the absolute value of agreement in step count for two-three-year olds (99.2 ± 4.6%) and 4-5-year olds (100.0 ± 4.4%). In this study, the use of percent agreement therefore rates as “excellent,” and the evidence of criterion validity for continuous walking is “good” which is further strengthened by the large sample size. Criterion validity for measurement of HPA cannot be determined based on this study. A further two studies assess the use of the StepWatch in children with CP [24] and DMD [25] but do not report any statistical methods or results which was rated “poor,” and criterion validity for these populations was therefore “indeterminate.”

Bland-Altman plots [32] and percent of activity laps which were detected were used to assess criterion validity in the combined study of the Minimod and AMP by Kuo et al. [23] and due to this would have scored “poor” on the COSMIN checklist [19]. The lack of a specific index to summarise the degree of agreement is a limitation of the Bland-Altman technique, and inferences about the estimate cannot be performed [33]. A strength of the Bland-Altman technique is that it produces a meaningful graph and computes the confidence limits from the paired differences between the criterion method (manual step count or meters walked) and the same variables measured by the Minimod [33]. This provides us with the ability to assess both accuracy and precision of measures and allows a comparison between groups and therefore still allows a thorough assessment of the agreement between methods and can be rated as an “excellent” statistical method. The Minimod performed well in continuous walking trials compared to measured length and direct observation of steps (mean difference = −0.4 m/−0.4 steps, 95% limits of agreement = −4.7 to 4 m/−4.1 to 3.3 steps) but showed a larger random error for activity lap walking (mean diff. = −2.3 m/−0.4 steps, 95% LoA = −27.9 to 23.3 m/−87.8 to 10.4 steps) [23]. The Minimod only detected walking activity in 19–37% of stair walking trials (ascent/descent) [23]. In TDC, the Minimod performed well for continuous walking trials and poor in structured activity laps (see Table 2); however it performed better for detecting stair walking (84%) [23]. The AMP showed greater underestimation and random error in continuous walking trials (mean diff. = −4.8 m/−3.5 steps, 95% LoA = −20.1 to 10.5 m/−16.9 to 10 steps) which increased with increasing distance walked [23]. The AMP performed better than the Minimod in the structured activity lap walking trials, but still showed considerable bias difference and large random error in this trial (mean diff. = −3.6 m/−11.2 steps, 95% LoA = −19.2 to 12.0 m/−40 to 17.7 steps), however detected more of the stair climbing trials (85%) [23]. Results were similar for TDC (see Table 2) [23]. Due to the lack of an index, a specific cut-off for what classifies as a “good” result cannot be set; however the results indicate that the Minimod has “good” accuracy and precision for continuous walking. When it comes to more free-living type activities such as the structured walking trial and stair walking, the performance of the Minimod is “poor.” The AMP showed “poor” precision and accuracy in both walking trials, however detected more stair trials. As the focus of this paper is the ability to measure free-living activities, both monitors score “poor” for evidence of criterion validity for measuring HPA.

A Pearson correlation coefficient was used to assess agreement between the EE measured by the IDEEA and the criterion method of indirect calorimetry using the Cosmed [26]. A Pearson correlation is considered an “excellent” statistical method by the COSMIN [19]. For the daily activities trial, total energy expenditure was used as the outcome measure. For comfortable and fast treadmill walking and stair climbing trials, energy expenditure rate (kcal/minute) was used. For children with CP and TDC the correlation between measurement outputs was “good” by COSMIN standards during all activities (CP: *r* = 0.70–0.88; TDC: *r* = 0.74–0.97, *P* ≤ 0.05) [26]. A limitation of the Pearson correlation coefficient is that it only measures precision not accuracy and is therefore not a true measure of agreement [33]. This is demonstrated by the authors of this paper as the IDEEA significantly overestimated energy expenditure during the series of everyday activities and during comfortable treadmill walking both in children with CP and TDC, and during fast treadmill walking in TDC (paired *t*-test, *P* < 0.01) [26]. During fast treadmill walking in children with CP and during stair walking in both groups, measured energy expenditure did not differ significantly between methods (paired *t*-test, *P* > 0.01) [26]. A limitation of the *t*-test is that it will only show a significant difference if there is a systematic constant difference between two values, not if there is a systematic proportional difference as the paired difference will end up close to zero [34]. A Bland-Altman plot or a regression analysis would provide a better identification of the nature of any systematic differences in the EE estimation of the IDEEA. The present energy expenditure calculations used in the IDEEA software do not accurately assess energy expenditure, and the clinimetric properties of the IDEEA are therefore considered “poor.” Authors suggest the good correlation between the IDEEA and the Cosmed indicates it can be used as a clinical follow-up tool for quantitative evaluation of efficacy of treatment interventions. As the IDEEA appears to perform better at higher intensities, systematic differences may exist which may lead to inaccurate conclusions. The reliability of EE measurement by the IDEEA was assessed in children with CP (*n* = 12) [26] using a Pearson's correlation coefficient and a paired *t*-test. Authors aimed to assess “agreement” between repeated measures as discussed in de Vet et al. [35]. This study therefore scored “poor” for choice of statistical method as measures can significantly correlate despite being significantly different, and the inability of a *t*-test to detect systematic proportional differences as discussed previously still applies [33, 34]. Agreement between repeated measures of EE using the IDEEA is therefore “indeterminate” based on this study. 

Authors of the StepWatch study in children with DMD [25] used a heart rate monitor to assess its concurrent validity and received a “good” rating for statistical method as they used a Pearson correlation coefficient but did not report standard deviation. Evidence of concurrent validity was rated poor as *r* < 0.70 (CP: *r* = 0.295; TDC: *r* = 0.477; *P* < 0.05).

### 3.3. Clinical Utility

Clinical utility was assessed for the four identified measures and is summarised in Table 2. The Minimod and StepWatch require individual calibration and software for analysis. The StepWatch is expensive (cost not available for Minimod). The rich data collected by the StepWatch and Minimod allows measurement of intensity, frequency, and duration of walking activity. The units are both small and unobtrusive and have battery lives of seven days for the Minimod and up to six weeks for the StepWatch, depending on settings. This makes them both feasible tools for the measurement of habitual walking activity. In the event that data collection is delayed once the device is provided to the child's parents, having a battery life of more than seven days, and therefore not requiring recharging is a strength of the StepWatch as there is less likelihood of loss of data. The Minimod is worn around the waist centred at the lower lumbar spine, while the StepWatch is worn on the ankle in a small cuff. Consistency of placement for repeated and all-day wear may be easier to achieve with an ankle placement which would reduce measurement error due to inconsistent placement. Both pedometers were considered to have good clinical utility. The AMP is small, worn around the ankle, does not require calibration or software analysis, and has a good battery life; however it only measures total step count or meters walked per wear period. The AMP was considered to have good clinical utility. The IDEEA consists of a data recorder worn on the waist with 5 individual sensors attached to the chest, thighs, and under each foot connected by wires to allow measurement of postures and energy expenditure, and it only has a battery life of 60 hours. Both of these factors limit the IDEEAs utility in the measurement of HPA in young children.

### 3.4. Level of Evidence

A summary of the quality of evidence for criterion validity is provided in Table 3. Two studies assess the Minimod in children with CP and TDC in samples which include children under the age of six. Both studies were rated “good” for study design and “excellent” for statistical method. They provided “good” evidence of the ability of the Minimod to accurately measure continuous walking. One of these studies [23] also used free-living type activities, and the Minimod displayed “poor” accuracy and precision in this setting. The AMP was assessed in one study of “good” study design quality which used an “excellent” statistical method, though displayed “poor” accuracy and precision in both continuous walking and free-living type activities.

Four studies assess the StepWatch in samples which included children under the age of six. Reporting of study protocol and statistical method was “poor” in three of these studies. The fourth study was of “good” quality and used an “excellent” statistical method. It provided “good” evidence of the ability of the StepWatch to accurately measure continuous walking in TDC. The ability of the StepWatch to accurately measure free-living activities cannot be determined based on this study. The ability of the IDEEA to measure EE was assessed in one study of “good” study design quality which used an “excellent” statistical method; however the IDEEA displayed “poor” accuracy and precision in both free-living type activities and continuous walking trials.

## 4. Discussion

This paper has systematically reviewed the clinimetric properties (validity, reliability, and clinical utility) of four measures of HPA in children with a motor disability which included children aged less than six years. A precise and accurate measurement of the daily physical activity levels in this population will allow researchers to investigate the dose-response relationship between HPA and health outcomes. It will also allow the assessment of the efficacy of interventions aimed at increasing HPA in terms of establishing the dose and distribution of treatment necessary to achieve worthwhile results in the long term. For clinicians, an accessible, precise, and accurate measurement tool would allow the identification of children with low levels of HPA and in turn the assessment of the effectiveness of prescribed interventions. 

Promising measures of HPA have been assessed in young children with motor disabilities, and the methodological quality of the papers was good to poor for study design and excellent to poor for statistical methods. A limitation of the findings of this paper is that only two studies of overall “good” methodological quality assessed children while undertaking free-living type activities. Under these conditions, both activity monitors displayed poor accuracy and precision [23, 26]. Two activity monitors displayed “good” accuracy during continuous walking [23, 28]. This relates more to the ICF definition of walking “capacity” rather than habitual walking activity (i.e., how many steps does a child need to walk a set distance in an ideal environment compared to how many steps do they take during the day in a variety of settings and intensities). Another limitation is that studies included in this paper assessed children across a wide age range (4–18 years), with no studies of children with motor disabilities exclusively focusing on the under six-year age group which limits this review's ability to make specific recommendations for this age group. The proportion of children aged less than six in the study samples ranged from 5 to 43% for the studies which provided a breakdown of their sample [22, 23, 26, 27]. The use of the COSMIN checklist for the assessment of methodological quality together with quality rating criteria of measurement properties is also a possible limitation. The COSMIN checklist was developed for assessing HR-PROs and does not have established psychometric properties in assessing objective physical activity measurement tools, and the quality rating criteria were not developed based on consensus. This issue was minimised by being guided by a systematic review of activity monitors in TDC for the assessment of the somewhat ambiguous “flaws in design or methods” item used in the COSMIN (Box H, item 5) [19] and by providing an in-depth appraisal of the statistical methods. 

The current Australian physical activity guidelines recommend children aged from 1 to 5 years engage in active play for at least three hours per day without specific intensity recommendations [2]. This recognises the sporadic and intermittent nature of young children's activity patterns and places demands on the measurement tools used to assess HPA in this population. Firstly they have to be able to record activity as a measure of time, and secondly they need to accurately recognise “active play” behaviours. The accelerometer-based pedometers StepWatch and Minimod have the ability to collect the most complete information on habitual walking activity as they record steps within an epoch of time which can be downloaded onto a computer to visualise an activity pattern. They allow the assessment of intensity (e.g., steps/min), frequency (e.g., number of walking bouts), and duration (e.g., length of walking bouts). The validation protocols of the included studies primarily used structured walking activities which may not accurately represent the way young children move in active play, and therefore it cannot be assumed activity monitors that do well in continuous walking trials would have done equally well had the children's steps been counted during free-living play. This was demonstrated in the assessment of the Minimod by Kuo et al. [23]. In typically developing children, activity monitors are usually validated by direct observation of free play to circumvent this issue, and validation studies typically use a narrower age range to control for differences in motor skills [36, 37].

 Children with a motor disability may not be able to walk but instead use a range of other methods of ambulation such as crawling, cruising, rolling, bottom shuffling, walking aids, or wheelchairs. Those who are able to walk may have different gait patterns than typically developing children [14]. This could explain why the accelerometer Minimod, which relies on recognising gait patterns to count steps, had a lower rate of activity detection in children with CP (19–97%) than in typically developing children (84–100%) [23]. Other measurement tools such as accelerometers, which report raw activity counts per epoch of time, bypass this limitation but are yet to be validated in young children with a motor disability. A systematic review of measures of HPA in TDC preschoolers identified the ActiGraph (Shalimar, FL, USA) accelerometer as having the best clinimetric properties in this population. Similarly, the systematic review of HPA measurements in adolescents with CP by Clanchy et al. [16] identified accelerometers as the most comprehensive measure of HPA patterns despite the limited evidence available, and the ActiGraph has since been validated in adolescents with CP [38, 39]. Studies of doubly labelled water [40, 41], the Compendium for Physical Activity Questionnaires [3] and ActiGraph in infants at risk of neurodevelopmental delay [42], did not meet inclusion criteria as their clinimetric properties for the measurement of HPA in children with motor disabilities aged less than six years had not been assessed. 

## 5. Conclusion

This systematic review identified four measures of HPA with evidence of clinimetric properties in study samples which included children aged less than six with motor disabilities. Only a very small number of studies assessing activity monitors in this population are available, and none of the studies focus exclusively on children aged less than six years. The pedometers StepWatch and Minimod are the most comprehensive measures of habitual walking activity utilised in the current literature. While both demonstrate good accuracy for step count during continuous walking, only the Minimod has been tested during conditions which included walking trials other than continuous walking, and it performed poorly during these conditions. It is possible the ankle placement of the StepWatch would allow a more accurate assessment of free-living walking activities in children with motor disabilities but this is yet to be demonstrated. Pedometers are only suitable as an estimate of HPA for children with high functional capacity as children's HPA patterns are likely to consist of a progressively smaller proportion of walking as the severity of their impairment increases. In the most severely impaired children walking activity is completely nonexistent. Further research is needed to ascertain the clinimetric properties of activity monitors available for measuring HPA in young children with motor disabilities, and testing protocols should include a range of activities and ideally direct observation of free play. This will enable an understanding of the HPA patterns of children with motor disabilities across the spectrum of functional capacity for clinicians and researchers alike.

## Figures and Tables

**Table 1:** Characteristics of studies describing measures of habitual physical activity.

Brand	*n*	Boys	Population			Protocol			
			Mean age (range)	Children aged ≤5 years (*n*)	Motor disorder	Reference	Setting (measurement units)	TDC	Study
								reference group	
Minimod^a^	17	—	10 yr 6 mo (4–16 yr)	~2	CP^1^	DO	Continuous walking, structured activity lap, and stair climbing (steps and meters walked)	*n* = 19, 4–16 yr	Kuo et al.[23]
	20	—	5–16 years (−)	1	CP^2^		Self-paced walking (steps and meters walked)	*n* = 20, 3–16 yr	Brandes et al. [22, 27]

AMP^b^	20	13	10 yr 6 mo (4–16 yr)	~2	CP^1^	DO	Continuous walking, structured activity lap, and stair climbing (steps and meters walked)	*n* = 20, 4–16 yr	Kuo et al. [23]

StepWatch^c^	16	16	9 yr 2 mo (5–13 yr)	—	DMD	DO	Slow/fast self-paced walking (steps) + 3-day HR and pedometer (steps and HR)	*n* = 21, 5–13 yr	McDonald et al. [25]
	27	22	−(4–18 yr)	—	CP^3^	DO	Treadmill walking at different speeds (steps)	*n* = 27, age matched	Stevens et al. [24]
	60	30	−(2-3 yr)	60	TDC	DO	Self-paced walking (steps)	n/a	Bjornson et al.
	62	31	−(4-5 yr)	62	TDC	DO	Self-paced walking (steps)	n/a	[28]

IDEEA^d^	20	10	−(5–11 yr)	—	TDC	DO	Self-paced walking and running (steps)	n/a	Song et al. [29]
	21	—	−(4 yr–10 yr 1 mo)	9	CP^1^	IC + HR	Series of everyday activities, walking on treadmill, climb staircase (energy expenditure kcal/min)	*n* = 7, 5 yr 8 mo–8 yr 6 mo	Aviram et al. [26]

^a^Minimod, Dynaport, McRoberts BV, Hague, Netherlands; ^b^AMP: Activity Monitoring Pod 331, Dynastream Innovations, Alberta, Canada; ^c^StepWatch, Orthocare Innovations, WA, USA, ^d^IDEEA: Intelligent Device for Energy Expenditure and Activity, Minisun, CA, USA; CP: cerebral palsy; GMFCS: Gross Motor Function Classification System; DMD: Duchenne muscular dystrophy; TDC: typically developing children; DO: direct observation; IC: indirect calorimetry; HR: heart rate monitor; ^1^GMFCS I–III; ^2^GMFCS not specified, able to walk; ^3^GMFCS I–II; (—): not reported.

**Table 2:** Evidence of criterion or construct validity, reliability and utility.

Measure	Utility	Study	*n*, age (years), disability	Criterion validity	Construct validity	Reliability
	Cost not available	Kuo et al. [23]	*n* = 17, 4–16, CP	Mean difference ± 2SD: (−3.3 ± 2.2)–(8.9 ± 2.5) m; (−38.7 ± 49.1)–(−1.0 ± 1.7) steps; rate of activity detection: 19–97%	n/a	n/a
	Calibration and analysis software		*n* = 19, 4–16, TDC	Mean difference ± 2SD (−0.6 ± 5.2)–(7.5 ± 2.8) m; (−57.4 ± 67)–(−1.0 ± 2.0) steps; rate of activity detection for stair ascent and descent 84–100%		
Minimod	Rich data collection			Agreement_steps_ = 98.9% (range: 94.1–101.8%)		
		Brandes et al. [22, 27]	*n* = 19, 5–16, CP	Agreement_distance_ = 101% (range not reported)		
	Feasible HPA measure			Agreement_steps_ = 99.6 ± 0.6% (range: 98.5–101.5%)	n/a	n/a
			*n* = 20, 3–16, TDC	Agreement_distance_ = 100.6 ± 3.3% (range: 93–106.7%)		
				Agreement_time_ = 101.3 ± 2.8% (range: 94.5–106.6%)		

AMP	Cost not available No calibration Analysis software	Kuo et al. [23]	*n* = 20, 4–16, CP	Mean difference ± 2SD (−4.8 ± 15.3)–(1.3 ± 2.5) m; (−11.2 ± 28.8)–(−3.5–13.4) steps; rate of activity detection 85–95%	n/a	n/a
	Only total steps and meters walked Feasible HPA measure		*n* = 20, 4–16, TDC	Mean difference ± 2SD (−2.5 ± 7.1)–(0.7 ± 1.0) m; (−4.4 ± 14.5)–(−1.3 ± 1.6) steps; rate of activity detection: 92–100%		

	Expensive (unit: $500, software: $1500)	McDonald et al. [25]	*n* = 16, 5–13, DMD	Authors state “no difference between observed and measured”; no statistical measure reported	*r* = 0.295, *P* < 0.05	
			*n* = 20, 5–13, TDC		*r* = 0.477,	n/a
	Calibration and analysis software				*P* < 0.05	
StepWatch		Stevens et al. [24]	*n* = 27, 4–18, CP	Authors state “readjusted until all valid step activity recorded”; no statistical measure reported	n/a	n/a
	Rich data collection		*n* = 27, 4–18,TDC			
		Bjornson et al. [28]	*n* = 162, 2–5, TDC	Agreement = 99.2 ± 4.6% (2-3yr); Agreement = 100.0 ± 4.4% (4-5yr)	n/a	n/a
	Feasible HPA measure	Song et al. [29]	*n* = 20, 5–11, TDC	Walking: *r* = 0.97; Running: *r* = 0.96; (*P* < 0.05); measurement error 3 ± 1%	n/a	n/a

IDEEA	Cost not available Poor battery life (60 hrs) Poor utility in HPA research	Aviram et al. [26]	*n* = 21, 4–10, CP	*r* = 0.72 (*P* < 0.001); difference means (*t*-test): *P* = 0.000		Test-retest
					n/a	*r* ≥ 0.995;
			*n* = 7, 5–8, TDC	*r* = 0.88 (*P* < 0.05); difference means (*t*-test): *P* = 0.002		Difference mean:
						*t*-test: *P* = 0.33

CP: cerebral palsy; DMD: Duchenne muscular dystrophy; TDC: typically developing children; AMP: ambulatory monitoring Pod; IDEEA: Intelligent Device for Energy Expenditure and Activity; n/a: not assessed.

**Table 3:** Evidence of criterion validity.

Measure	Study	Study design	Statistical method	Criterion validity for HPA
Minimod	Kuo	++	+++	−
	Brandes	++	+++	u/a
AMP	Kuo	++	+++	−
IDEEA	Aviram	++	+++	−
StepWatch	Stevens	−	u/a	u/a
	McDonald	−	u/a	u/a
	Song (TDC only)	−	−	u/a
	Bjornson (TDC only)	++	+++	u/a

IDEEA: Intelligent Device for Energy Expenditure and Activity; TDC: typically developing children; (+++): excellent; (++): good; (+): fair, (−): poor, (u/a): unable to assess/indeterminate.
